# Pertussis Epidemiology in the Autonomous Province of Vojvodina, Serbia, 1948–2023

**DOI:** 10.3390/vaccines12050525

**Published:** 2024-05-10

**Authors:** Mioljub Ristić, Snežana Medić, Vladimir Vuković, Smiljana Rajčević, Marko Koprivica, Jelena Banjac, Stefan Ljubičić, Vladimir Petrović

**Affiliations:** 1 Department of Epidemiology, Faculty of Medicine, University of Novi Sad, 21000 Novi Sad, Serbiavladimir.petrovic@izjzv.org.rs (V.P.); 2 Institute of Public Health of Vojvodina, 21000 Novi Sad, Serbia; 3 Public Health Institute Subotica, 24000 Subotica, Serbia

**Keywords:** pertussis (whooping cough), epidemiology, surveillance, vaccination, Vojvodina, Serbia

## Abstract

Pertussis continues to be a significant public health concern. We aimed to examine the epidemiological characteristics of pertussis in Vojvodina, which accounts for almost a third of Serbia’s population. Our aim was to determine the overall and age-specific incidence and mortality rates of pertussis in Vojvodina from 1948 to 2023, as well as the coverage of immunization against pertussis from 1960 to 2023. In the period 1948–2023, 42,259 cases of pertussis were reported. Following the introduction of the DTwP vaccine (1960) in Serbia, the reported incidence of pertussis began to decline. In 2001, for the first time since introduction of pertussis surveillance in Vojvodina, no pertussis cases were reported. Since 2012, the reported incidence of pertussis has once again increased, and peaked (41.1/100,000) in 2023, approaching the incidence rates recorded shortly after the introduction of DTwP vaccine. A shift in the age profile of pertussis from children aged 0–6 years to school-aged children (7–14 years) occurred between 2012 and 2023, when 48.3% of pertussis cases occurred in this age group. Although the incidence rates of pertussis among individuals aged 20 years and older were significantly lower than among younger age groups, there is evidence of an increasing trend in pertussis cases, particularly among those aged 40–49 years, since 2012. Based on the findings of this study, it is imperative to introduce additional booster doses of the aP vaccine for individuals aged 14 years, along with implementing maternal immunization strategies targeting women of childbearing age.

## 1. Introduction

Pertussis (whooping cough) is a highly contagious infectious disease that affects the respiratory tract of patients, with the potential complications, including death. Worldwide vaccination against pertussis began in the 1950s. The widespread implementation of immunization against pertussis has resulted in a significant reduction in pertussis-related childhood mortality. Pertussis can present as a severe condition in infants, leading to significant morbidity. However, in adolescents and adults, it commonly manifests as a mild respiratory illness, and frequently goes unrecognized [1]. Despite the fact that pertussis is a vaccine-preventable disease, it is endemic worldwide, with cyclical epidemics every 3–5 years [1,2]. In addition, pertussis remains among the least-controlled vaccine-preventable diseases in European Union/European Economic Area (EU/EEA) countries, as well as across the broader European region, including Serbia [3]. One of the possible reasons for the re-emergence of pertussis worldwide in the last decade is waning immunity after immunization, highlighting the need to determine the optimal timing for booster doses [4,5].

The aim of this paper was to observe the epidemiology of pertussis in Vojvodina (northern Serbian Province) between 1948 and 2023, with the ultimate goal of updating public health recommendations and providing evidence for the urgent implementation of additional booster doses of the pertussis vaccine in our country.

## 2. Materials and Methods

### 2.1. National Reporting System in Serbia

In Serbia, the official notification of pertussis, based on clinical criteria, has been mandatory since 1948. Since 2012, in the Autonomous Province of Vojvodina (Vojvodina), an improved pertussis surveillance was introduced based on the Global Pertussis Initiative (GPI) recommendations (specific clinical case definitions and laboratory confirmation of pertussis) [6,7].

The database for this analysis included all reported cases of pertussis, as well as deaths due to pertussis from 1948 to 2023 in Vojvodina (≈1.7 million inhabitants, which corresponds to almost one-third of the population of Serbia), reported to the Centre for Disease Control and Prevention, Institute of Public Health of Vojvodina (IPHV), as part of the routine surveillance of communicable diseases.

Data from 1948 to 2011 were obtained through passive surveillance using the case definition of pertussis that was based on clinical diagnosis; i.e., according to a clinical assessment and the physician’s opinion, using a culture confirmation or without laboratory confirmation of pertussis, and the pertussis vaccination history and epidemiological data. In addition, due to issues with tracking, recognizing, and reporting, the number of pertussis cases during the period 1948–1953 was lower than in the second part (1954–1960) of the pre-vaccine era. Similar problems were recognized regarding the reported deaths related to pertussis in our territory [7,8,9]. In contrast to the data obtained by passive surveillance of pertussis from 1948 to 2011, data from 2012 to 2023 were collected based on the results of improved pertussis surveillance system, which has, so far, been introduced only in Vojvodina. This surveillance is case-based and relies on the GPI clinical case definitions of pertussis and the implementation of laboratory confirmation for pertussis [6].

### 2.2. National Immunization Program in Serbia

Mandatory vaccination against pertussis in Serbia started in 1960, and a whole-cell pertussis vaccine as a component of DTwP vaccine (along with diphtheria and tetanus toxoids) was introduced. This was followed by a significant reduction in the incidence of pertussis. The primary series of vaccination against pertussis is conducted with three doses of vaccines for all children starting at the age of 2 months, and should be completed by 6 months with a minimum interval of 4 weeks between subsequent doses. Revaccination is performed (first booster) one year after the third dose of vaccine from primary series. The second booster of the DTwP vaccine for children aged 4 years was introduced in 1981, and it was left out in 1985, and from 1986 to 2001, a monovalent pertussis vaccine was administered to children in the same age group [8,9,10].

In 2015, the DTwP vaccine was officially replaced by the DTaP-IPV-Hib vaccine, which combines protection against diphtheria, tetanus, pertussis, polio, and Haemophilus influenzae type b. DTaP-IPV-Hib vaccine was also available on the private market in Serbia, between 2001 and 2014. Following a period without a second pertussis booster dose in Serbia (between 2002 and 2021), a second booster against pertussis was reintroduced in 2022 in a combined diphtheria–tetanus–acellular pertussis–polio vaccine (DTaP-IPV) for children aged 6–7 years. Boosters against pertussis for adolescents, adults, pregnant women, or healthcare workers (HCWs) were introduced to the Serbian National Immunization Program, but the vaccines are not currently available [7,10,11].

We collected and analysed the data on pertussis immunization coverage with three doses of pertussis containing vaccines during the first year and revaccination during the second year of life in Vojvodina from 1960 to 2023.

### 2.3. Laboratory Procedures

Samples from patients suspected of pertussis in the period 2012–2023 were tested at the Centre for Microbiology, IPHV, Novi Sad. According to the GPI propositions [6], the choice of sample and laboratory method (PCR or serology tests) was contingent upon the duration of cough symptoms and the patient’s age, as elaborated previously [7].

### 2.4. Statistical Analysis

Data on pertussis incidence and mortality rates in Vojvodina during the period 1948–2023, along with DTw/aP3 vaccination and DTw/aP revaccination coverage rates, were obtained from annual reports of the Centre for Disease Control and Prevention, IPHV, spanning the entire period. Incidence and mortality rates of pertussis were calculated using the annual number of registered cases as the numerator and the population of Vojvodina, according to the census, as the denominator [12], multiplied by 100,000. The case fatality rate was expressed as the proportion of pertussis-related deaths to all reported pertussis cases. Administrative immunization coverage data represent the number of vaccine doses administered to the designated population or the number of immunized individuals within that population within one calendar year. The target population groups differ among countries, and are contingent upon the national immunization schedule. Coverage estimates derived from the administrative method may be subject to bias stemming from inaccurate numerators or denominators. [13]. Despite this disadvantage, the administrative method was utilized to present the annual coverage of immunization (DTw/aP3 vaccine and DTw/aP revaccine) as the annual percentage of immunized children out of the total number of children in birth cohorts in Vojvodina.

All annual data were collected in collaboration between the IPHV and six IPHs located in each of six districts of Vojvodina, based in Subotica, Sombor, Pančevo, Sremska Mitrovica, Kikinda, and Zrenjanin.

## 3. Results

**Figure 1:**
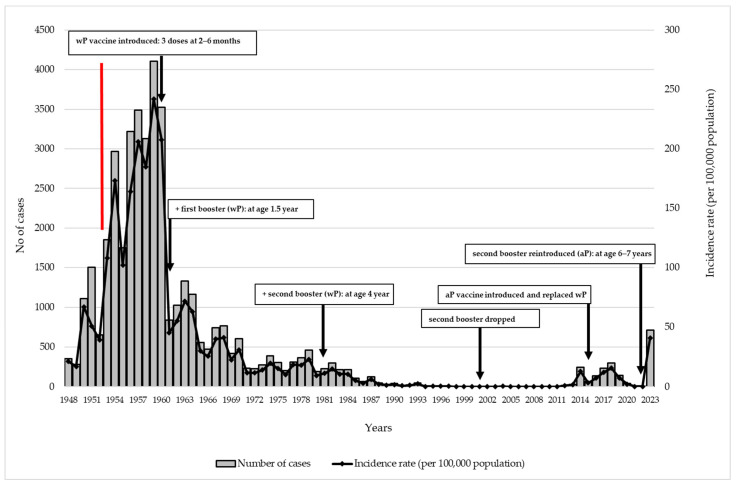
Reported cases and incidence rate of pertussis, per 100,000 population, in Vojvodina, 1948–2023. Legend: wP: whole-cell pertussis vaccine; aP: acellular pertussis vaccine; the red vertical line indicates the separation between the period of introduction of surveillance and its wider implementation across Vojvodina.

Figure 1 summarizes the number of cases and incidence rates of pertussis in Vojvodina during the 1948–2023 period, as well as the type of pertussis vaccine and year of its introduction.

In the pre-vaccine era, thousands of pertussis cases were reported every year, with the highest incidence rate (242.1/100,000 population) reported in 1959. After the introduction of the DTwP vaccine, the reported incidence of pertussis sharply declined, but remained above 50/100,000 during the 1960s, with epidemics in 1963–1964 and 1967–1968. In the 1970s and 1980s, the incidence of pertussis continued to decline, ranging from 1.4/100,000 (in 1989) to 31/100,000 (in 1970) and, during the 1990s, only sporadic pertussis cases were registered. In 2001, there were no reported pertussis cases, and between 2002 and 2011, only 14 cases of pertussis were recorded. Since 2012, the reported incidence of pertussis has increased again, and peaked in 2014, 2018, and 2023. From 2012 to 2023, a total of 1949 pertussis cases were reported. In 2023, the incidence rate of pertussis has reached 41.1/100,000, approaching the incidence observed in 1961 (45.4/100,000), one year after the introduction of vaccination against pertussis.

**Figure 2:**
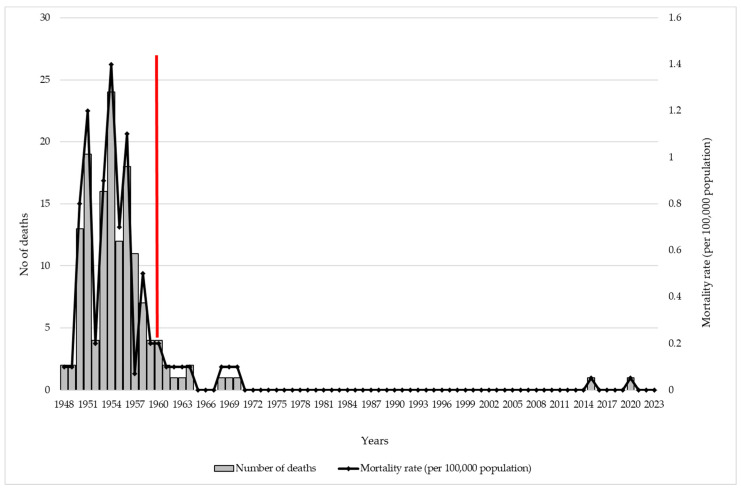
Reported deaths and mortality rate of pertussis, per 100,000 population, in Vojvodina, 1948–2023. Legend: the red vertical line indicates the separation between the period before and after introduction of immunization against pertussis in Vojvodina.

During the pre-vaccine era (1948–1960), a total of 136 deaths due to pertussis were registered, with the highest mortality rate reported in 1954 (1.4/100,000). In the vaccine period (1961–2023), a total of 11 deaths due to pertussis were notified. Most of them (n = 9) were registered in the period 1961–1970. The average mortality rate of pertussis in the pre-vaccine period was 0.6/100,000, while in the vaccine period, it was 0.01/100,000 (Figure 2).

Throughout the study period, the highest number (50) of deaths related to pertussis was recorded among infants under 1 year of age. More precisely, out of the total number of deaths due to pertussis in the period 1948–2023, 34%, 25.9%, and 20.4% were recorded among infants, followed with the children aged 1 year, and those aged 2 years, respectively. Case fatality rates in three mentioned ages were 1.03%, 0.62%, and 0.54%. Only one death was recorded among patients aged ≥ 10 years in 1950 (Table 1).

**Figure 3:**
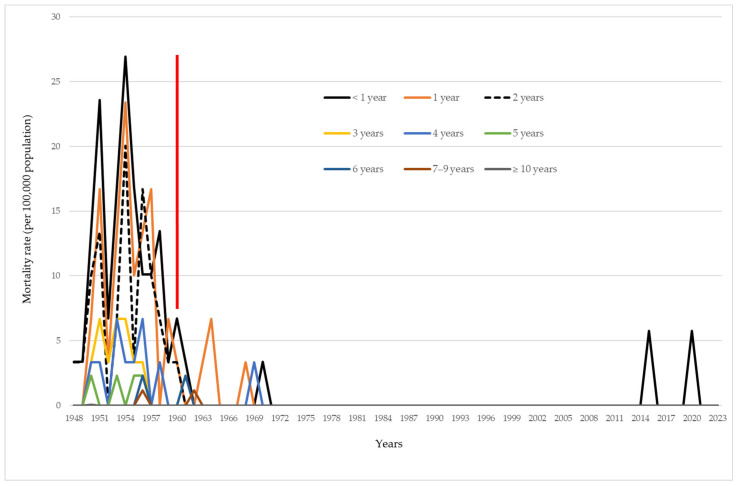
Mortality rate of pertussis, per 100,000 population, in Vojvodina, 1948–2023. Legend: the red vertical line indicates the separation between the period before and after introduction of immunization against pertussis in Vojvodina.

The highest age-specific mortality rates in infants aged under 1 year (26.9/100,000), children aged 1 year (23.4/100,000), and in children aged 2 years (20.1/100,000) were registered in 1954. Between 1971 and 2014, there were no registered deaths related to pertussis. In 2015 and 2020, deaths due to pertussis were registered in infants aged 2.5 months and 14 days, respectively (Figure 3).

During the observed period, a total of 42,259 cases of pertussis were reported. Out of total number of cases, 78.3% (33,070/42,259) were recorded in preschool-aged children (0–6 years of age). Between 2012 and 2023, a total of 1949 pertussis cases were registered. In 2023, for the first time, the number of pertussis cases (n = 385) among children aged 10–14 was 12.4 times higher than in infants under 1 year (n = 31).

Since 2012, when improved surveillance of pertussis was implemented, cyclical epidemics of pertussis were observed in 2014, 2017/2018, and 2023.

**Figure 4:**
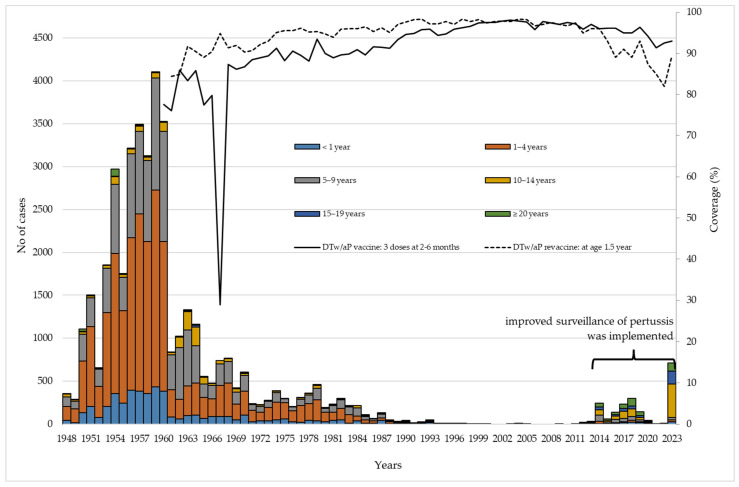
Number of pertussis cases by age (1948–2023), and DTw/aP immunization coverage (1960–2023) in Vojvodina.

Immunization coverage with primary series and revaccination against pertussis varied over the study period. Since the introduction of vaccination against pertussis, the lowest value (29%) of DTwP3 coverage was measured in 1967 and, thereafter, it continuously increased until 1990. It was consistently above 95% from 1990 to 2019. Coverage for DTaP3 between 2020 and 2023 ranged from 91% (in 2021) to 94% (in 2020). Coverage for the first booster reached ≥95% in 1974, and remained at that level until 2014. During the period of 2015–2023 it dropped and ranged from 82% (in 2022) to 93% (in 2015 and 2019). In the period of 2012–2023, the average coverage of the DTaP3 vaccine was 95%, and the average coverage of DTaP booster vaccine was 90% (Figure 4).

**Figure 5:**
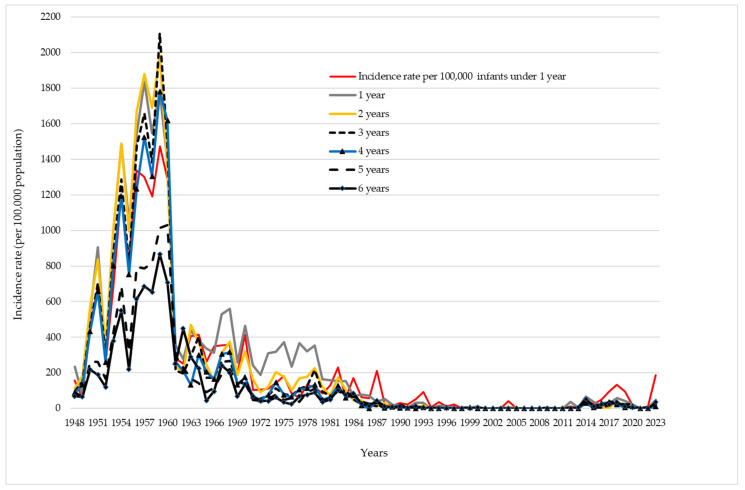
Incidence rate of pertussis, per 100,000 population, among preschool children in Vojvodina, 1948–2023.

The age-specific incidence rates of pertussis in preschool-aged children are shown in Figure 5. Between 1948 and 1959, the incidence rates of pertussis among infants under 1 year of age ranged from 64 to 1471 per 100,000 infants, but higher peaks of incidence rates were observed among children aged 1, 2, and 3 years. During the same period, the highest incidence rates of pertussis for children aged 1, 2, and 3 years were 1835.1/100,000 (in 1957), 1985.6/100,000 (in 1959), and 2109.2/100,000 (in 1959), respectively. Following the introduction of the pertussis vaccine, the incidence rates of pertussis dramatically declined in preschool-aged children. Between 2012 and 2023, there was only an increase in the incidence among the youngest population (infants aged under 1 year), but it was significantly lower than during the pre-vaccine period.

**Figure 6:**
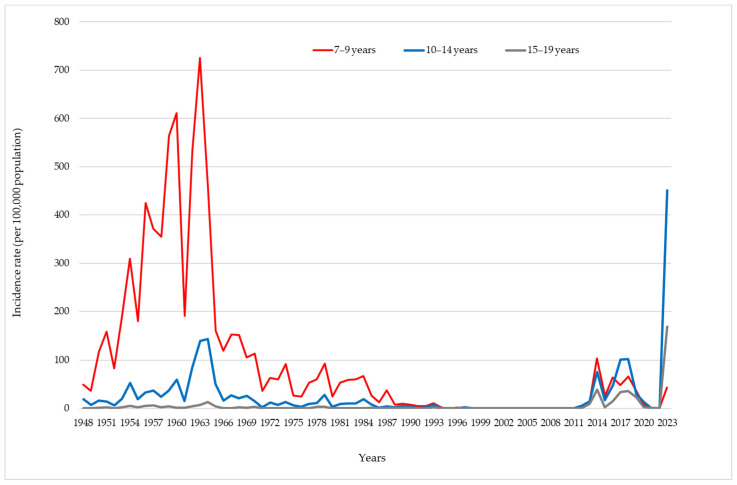
Incidence rate of pertussis, per 100,000 population, among school-aged children and adolescents (aged 7–19 years) in Vojvodina, 1948–2023.

Regarding the distribution of pertussis among children aged 7–9, 10–14, and 15–19 years, it is evident that pertussis was more frequently registered among children aged 7–9 years compared to the other two age groups. A higher incidence rate of pertussis in the 7–9 age group, compared to 10–14 and 15–19 years, was also registered during the vaccine period, with the peak of incidence rate (725.2/100,000) seen in 1963. Between 2012 and 2023, an increase in incidence across all three age groups was registered, but the higher rates were among children aged 10–14 and adolescents aged 15–19 years than among those aged 7–9 years (Figure 6). In 2023, the incidence rate of pertussis in the 10–14 age group was 450.8/100,000, marking the highest incidence rate within this age group throughout the entire study period. This rate was also 2.4 times higher than the incidence rate of pertussis among infants (186.3/100,000) in 2023.

**Figure 7:**
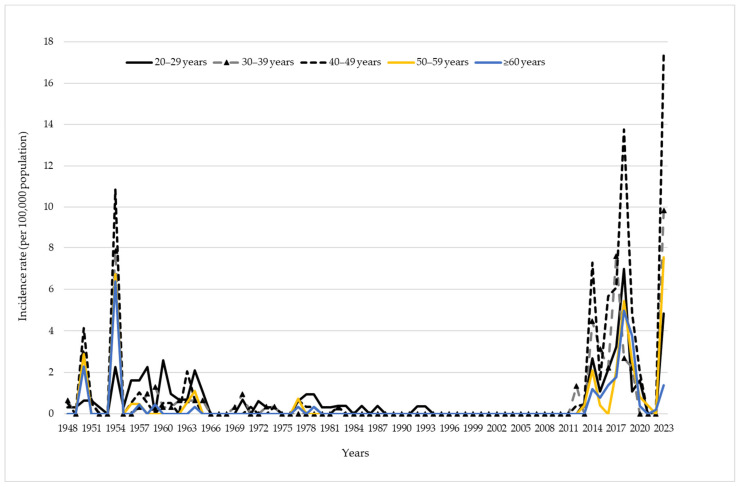
Incidence rate of pertussis, per 100,000 population, among persons aged 20 years and older in Vojvodina, 1948–2023.

Figure 7 summarizes the age-specific distribution of pertussis among adults aged 20 years and older. Although the incidence rates of pertussis in the adult population were significantly lower compared to younger individuals, after 2012, an increasing trend of pertussis was recorded across all observed age groups, especially among persons aged 40–49 years. In 2014, 2018, and 2023, incidence rates of 7.3, 13.8, and 17.4/100,000, respectively, were recorded for those aged 40–49 years. These respective rates were even higher than those observed during the pre-vaccine era.

## 4. Discussion

Before the widespread introduction of vaccination, pertussis was primarily considered a childhood disease. For example, in the USA, more than 95% of officially registered pertussis cases during the pre-vaccine period were in children under 10 years of age [14]. Following the development and implementation of immunization with vaccines containing a pertussis component, the incidence of the disease was significantly reduced. On the other hand, after the widespread introduction of immunization and achieving high coverage of vaccination against pertussis, the age distribution of the disease has shifted from infants to adolescents and adults [2,5,6,15,16]. The results of our study have also confirmed that the introduction of the pertussis vaccine and routine vaccination programs in Serbia in 1960 led to a decline in the overall incidence and mortality rates of pertussis. In 2001, forty-one years after the introduction of immunization against pertussis, no cases of pertussis were reported in Vojvodina. It is worth noting that the second dose of the pertussis vaccine was administered in Serbia between 1981 and 2001 [9], which had an additional effect on the optimal reduction of pertussis cases in our territory. However, due to insufficient diagnostic capabilities by PCR or serology until 2012, cases in older age groups remained unrecognized and, therefore, underreported.

In order to better assess changes in the epidemiology of pertussis over time, it is important to improve surveillance using clinical case definitions and laboratory diagnosis of pertussis [1,3,6]. Given that the officially reported number of pertussis cases may not accurately reflect the true extent of the disease, and considering that pertussis is often underrecognized, particularly among adolescents and adults, since 2012, the GPI case definition of pertussis concurrently with the increasing capabilities of laboratory testing and confirmation were implemented in Vojvodina, as previously described in detail [7]. Closely after the implementation of improved pertussis surveillance in Vojvodina, an increasing trend of pertussis incidence was recorded.

Findings of a comprehensive study conducted across 15 European countries between 2010 and 2017 suggests that pertussis immunization, whether recommended or mandated, along with the administration of boosters in adolescents, indeed impacts the incidence rate of pertussis. However, it does not alter the cyclic pattern of pertussis occurrence [17].

The purpose of vaccination against pertussis is to reduce the severity of and mortality related to pertussis [1]. The results of our study have shown a decline in number deaths related to pertussis in vaccine era. More precisely, total of 136 deaths related to pertussis were reported in the pre-vaccine period (1948–1960), while in the vaccine era (1961–2023), a total of 11 deaths due to pertussis were recorded in Vojvodina. After 1970, and after 45 years without registered deaths due to pertussis, the last cases of pertussis deaths registered in Vojvodina were one in 2015, and another in 2020.

The age distribution of pertussis is characterized by the disease occurrence, regardless of the patient’s age. Nevertheless, older children, adolescents, and adults typically present with less severe symptoms, whereas infants, pregnant women, and the elderly are at higher risk of experiencing pertussis complications. [18]. Additionally, infants (mainly those less than 3 months old) have the highest mortality rate [1,19]. The burden of this disease in Vojvodina was significant, in which the highest mortality rate was recorded among infants and 34% of the total number of deaths in the period 1948–2023 were recorded among the youngest age group. On the other hand, only one pertussis related death was recorded among patients aged ≥10 years.

In the context of the global and regional immunization profile for 2022, vaccine coverage of three doses of DTw/aP in the first year of life ranged between 85% and 86% in most countries. Additionally, many countries included a pre-school and an adolescent booster in their national immunization programs [20]. In addition, the global coverage of DTP (both DTwP and DTaP) ranged from 86–90% for the first dose, and from 81–86% for the third dose in the period from 2008 to 2021. The global coverage of the pertussis contained vaccines decreased in the period from 2020 to 2022, compared to the period 2012–2019, probably as a consequence of the COVID-19 pandemic [21]. Immunization coverage with the primary series and revaccination against pertussis in Vojvodina has been high during most of the study period. The lowest level of DTwP3 coverage (29%) was recorded in 1967, probably due to consequences of catastrophic floods in Serbia and the cessation of vaccine supplies [22]. Thereafter, DTw/aP vaccination and revaccination coverage continuously increased. During the COVID-19 pandemic years, coverage for the third dose vaccine against pertussis ranged from 91% (in 2021) to 94% (in 2020). Coverage for the first booster during the period from 2015 to 2023 ranged from 82% (in 2022) to 93% (in 2015 and 2019). Non-pharmaceutical interventions have helped curb the transmission of the COVID-19, but have also disrupted many health care services, including immunization. During the pandemic, vaccination appointments were often missed due to fear of visiting health facilities [23]. Consequently, global coverage with three doses of pertussis containing vaccines dropped from 86% in 2019 to 83% in 2020, as a result of public health crises caused by the pandemic [20,23].

Despite widespread coverage with DTw/aP4 or DTw/aP5 in many countries, pertussis remains endemic, with the peak age of infection seemingly transitioning from infants and young children to adolescents. There are at least two recognized reasons for this. First, high DTP4 coverage has led to a change in the age distribution of pertussis, characterized by a shift in the peak age of infection away from infants to young children. Second, the implementation of a booster dose administered at school entry age has additionally contributed to the transition of the peak age of pertussis infection towards adolescents and adults [24,25]. For instance, results from the sentinel surveillance study in Japan showed a change in the age-specific proportion of cases between 2000 and 2015. In 2001, 27% of the cases were aged 6–11 months and 3% were aged 20 years and older, while by 2010, the proportion of cases among patients aged 20 years and older increased to 48%, and those 6–11 months accounted for only 4% [26]. Similarly, we found that ~78% of pertussis cases in Vojvodina occurred among preschool-aged children (aged 0–6 years) in the period 1948–2023. A shift in the age profile of pertussis patients occurred between 2012 and 2023, when over 48% of cases were registered in the school-aged children (aged 7–14 years). In 2023, for the first time since the introduction of pertussis surveillance in Vojvodina, the number of pertussis cases among children aged 10–14 was higher (12.4 times) than among infants.

Despite the resurgence of pertussis in many countries, it remains an under-estimated disease worldwide. There are numerous reasons for this phenomenon, including the variability in case definitions of pertussis used worldwide, the diverse clinical presentations of disease across age groups, alterations in clinical characteristics of diseases due to immunization, limited access to laboratory confirmation of the disease, a low index of suspicion among many physicians, and the occurrence of mixed infections [6,7]. As a result of improved surveillance of pertussis in Vojvodina from 2012 to 2023, in parallel with an increase in the incidence of pertussis, the age-specific incidence peaked among children aged 10–14 and adolescents aged 15–19 years. In addition, during 2023, the incidence rate of pertussis among the 10–14 age group was the highest during the entire 76-year period.

However, despite the implementation of improved surveillance since 2012, we have provided evidence that many cases were still unrecognized. In the seroprevalence study conducted using serum samples collected between 20 January 2016, and 15 June 2017, from participants residing in the city of Novi Sad (the main administrative centre of Vojvodina), our findings indicate that the actual number of pertussis cases is significantly greater than those officially reported. Specifically, the prevalence of pertussis unrecognized infection within one year was 3.4%, equivalent to 9242 cases, while only 200 cases were officially registered (ratio 46:1). Interestingly, we have additionally identified unrecognized recent pertussis infections or reinfections following waning immunity among young or future parents, as well as among other elderly family members [27]. Due to the implementation of non-pharmaceutical measures against the COVID-19 pandemic (such as movement restrictions, closures of schools, kindergartens, and faculties, physical distancing, wearing properly fitted masks, practicing hand hygiene, and prohibiting visits to secondary and tertiary healthcare institutions as well as nursing homes) [28], there has been a decline in the number of officially registered pertussis cases. Namely, during 2020–2022 in Vojvodina, 44, 3, and 5 pertussis cases were recorded, respectively. In the same period, vaccination coverage for the DTaP3 vaccine was 94%, 91%, and 93%, while for DTaP revaccination in the second year of age, it was 87%, 85%, and 82%, respectively. After these three (2020–2022) years, similarly to the other settings [29,30], a sudden increase in the number of pertussis cases across all age groups in Vojvodina was recorded. Possible reasons for this include the lower circulation of SARS-CoV-2 in the population and, consequently, the relaxation or absence of measures focused on controlling the spread of respiratory infections; decreased coverage of pertussis immunization during the pandemic years; accumulation of a susceptible population who did not come into contact with *Bordetella pertussis* (*B. pertussis*) during the first three pandemic years; and regression of immunity in previously vaccinated individuals, as well as increased public awareness and laboratory testing for pertussis following the COVID-19 pandemic. Taking into account that pertussis has cyclical epidemics every 3–5 years, it is noteworthy that in the last decade, pertussis has appeared as an epidemic in Vojvodina in 2014, 2017/2018, and 2023. However, the highest number of cases was recorded in 2023, which is consistent with the aforementioned reasons. Finally, although the incidence rates of pertussis among individuals aged 20 years and older were significantly lower than among younger age groups, an increasing trend in pertussis cases has been observed in adult populations since 2012, particularly among those aged 40–49 years. A similar epidemiological situation was observed in neighbouring Croatia, where the number of pertussis cases in 2023 exceeded the annual number reported over the past 40 years [31]. Specifically, Croatia reported 6261 cases of pertussis from 1 January 2023 to 15 March 2024. Among these cases, 67% occurred in youths aged 10–19 years. Additionally, Denmark reported 1229 cases in 2023, marking the highest annual number reported since 2007 [32]. Owing to disparities in surveillance systems, diagnostic techniques, and reporting protocols, the reported incidence of pertussis has exhibited considerable variation across European countries over the last 15 years. Nonetheless, there is substantiated evidence indicating pertussis circulation among adolescents and adults in Europe. While pertussis-associated morbidity and mortality are highest in infants, individuals aged over 50 years are also at increased risk [24].

For example, in a hospital-based surveillance study conducted in Portugal between 2000 and 2015, 94% of hospitalized pertussis cases were infants under 1 year old, with a case fatality rate (CFR) of 0.8%. Conversely, among hospitalized adult cases of pertussis, the CFRs were 11.5% in patients aged 18–64 years and 17.4% for those aged over 65 years [33]. In our study, CFR was the highest (1%) among infants under 1 year, and during the 76-year period, only one death related to pertussis among adults was recorded (in 1950).

Numerous other factors contribute to the increasing incidence of pertussis globally, including the lower efficacy of the DTaP vaccine compared to the DTwP vaccine. Natural infection and immunization with the DTwP vaccine induce a strong T-helper 1 and Th17 cell response, whereas the DTaP vaccine is associated with the induction of Th1 and a higher Th2 response, which is not as effective against *B. pertussis* infection. Additionally, studies in a baboon model have shown that the acellular vaccine does not fully protect against pertussis infection, although it does prevent symptoms [24,30]. Other factors, such as waning immunity following acellular vaccination, strain changes in the presence of vaccine-induced immunity, local collections of unvaccinated and, therefore, susceptible children, the improvement of laboratory capacity for polymerase chain reaction (PCR) confirmation of *B. pertussis*, and the emergence of non-vaccine type strains, also contribute to the resurgence of pertussis [24,30,34,35,36].

The prevailing consensus suggests that traditional vaccination strategies alone are insufficient in effectively managing the resurgence of pertussis. Hence, there is a pressing need for novel, targeted prevention and control strategies that account for the evolving epidemic dynamics and transmission patterns of the disease [29,30]. Until such measures are implemented, the World Health Organization continues to recommend the use of whole-cell pertussis (wP) vaccines. Countries that have switched to acellular pertussis (aP) vaccines are encouraged to implement supplementary measures. These may include the introduction of additional booster doses, and strategies such as maternal immunization, as well as the “cocooning” strategy. Given that immunity against pertussis declines over time, maternal immunization demonstrates particular efficacy in preventing pertussis-related deaths among infants. This outcome is likely primarily attributed to the direct protection provided by the transfer of maternal antibodies, along with a reduction in the risk of transmission due to the decreased likelihood of peripartum pertussis in the mother. For individuals aged 7 years and older, vaccines containing only acellular pertussis components should be administered [1,30]. There is substantial evidence indicating that additional booster doses provide direct protection against pertussis within the targeted age groups [37,38,39]. Consistent with this, a comprehensive Norwegian study covering the period between 1998 and 2019 has demonstrated that the introduction of booster vaccines for 7–8-year-olds and 15–16-year-olds influences a decrease in the incidence rate of pertussis, as a result of direct protection among the targeted age groups. Additionally, as a result of the introduction of booster doses for 7–8 and 15–16-year-olds, the authors of this study also found a decrease in pertussis incidence among adults (indicating indirect protection), but not among infants [40].

The resurgence of pertussis has been noted across both developed and developing countries globally [17,36,41]. However, despite evidence indicating an increasing number of pertussis cases in Vojvodina, the incidence rates remain significantly lower than those observed before the introduction of immunization, except in adults.

This study had some limitations. First, due to the passive nature of surveillance, there is a likelihood of underreporting pertussis cases in Vojvodina, especially during the period from 1948 to 2011, and particularly during the initial phase (1948–1953) of pertussis surveillance in Vojvodina. Diagnosis of pertussis in passive surveillance relies on clinical suspicion in the absence of laboratory confirmation, resulting in many cases in both children and adults going undiagnosed. Second, we did not collect data on individual patients’ vaccination history against pertussis, and there was only annual immunization coverage by cohorts vaccinated against pertussis in Vojvodina. Additionally, there was no annual coverage of the second booster dose for children aged 4 years during the period from 1981 to 2001. Given the declining trend of annual pertussis incidence rates during this period, it appears that the coverage of revaccination was high. Third, hospitalization rates were not calculated, due to the insufficient data about hospitalization over the study period. Fourth, data on the time between pertussis symptom onset and date of sampling in period from 2012 to 2023 were incomplete, as well as data on negative test results. Given the recommendation that serology should not be used during the first year after pertussis vaccination [6,7,25,42], and the lack of vaccination history data for the cases, the possibility of false positive serological results among vaccinated infants cannot be ignored. Despite the potential limitations outlined above, we posit that they did not significantly influence the main findings of our study, particularly given the long follow up period of pertussis surveillance in Vojvodina.

## 5. Conclusions

Despite the introduction of immunization and decades of high vaccination coverage, pertussis in Vojvodina remains endemic, especially in adults, and reported cases have been increasing.

Yet, over the last decade of the study period, we observed an increase in incidence rates of pertussis in all age groups, particularly among 10–14-year-olds, in parallel with a decrease in immunization coverage. Furthermore, we noted a rise in officially reported pertussis cases among individuals aged 20–49 years. Based on these findings, it is imperative to enhance and maintain immunization coverage with pertussis-containing vaccine during the first and second years of life, as well as before entry school, and to introduce an additional booster dose of the aP vaccine for individuals aged 14 years. Additionally, maternal immunization strategies targeting women of childbearing age should be implemented, and boosters in adults should be considered.

## Figures and Tables

**Table 1:** Number of reported cases, deaths due to pertussis and case fatality ratio (CFR) of pertussis in Vojvodina by age, 1948–2023.

Age/Age Group	Variables	Period 1948–1960	Period 1961–2023	Total
0	No of cases/deaths	3243/46	1626/4	4869/50
	CFR	1.42	0.24	1.03
1	No of cases/deaths	4091/34	2063/4	6154/38
	CFR	0.83	0.19	0.62
2	No of cases/deaths	4221/30	1299/0	5520/30
	CFR	0.71	-	0.54
3	No of cases/deaths	3825/10	984/0	4809/10
	CFR	0.26	-	0.21
4	No of cases/deaths	3525/9	934/1	4459/10
	CFR	0.26	0.11	0.22
5	No of cases/deaths	2912/4	985/0	3897/4
	CFR	0.14	-	0.10
6	No of cases/deaths	2313/1	1049/1	3362/2
	CFR	0.04	0.09	0.06
7–9	No of cases/deaths	2996/1	2744/1	5740/2
	CFR	0.03	0.04	0.03
≥10	No of cases/deaths	802/1	2647/0	3449/1
	CFR	0.12	-	0.03
Total	No of cases/deaths	27,928/136	14,331/11	42,259/147
	CFR	0.49	0.08	0.35

Legend: CFR: case fatality ratio.

## Data Availability

The data that support the findings of this study are available from the corresponding author upon reasonable request.

## References

[1] World Health Organization (2016). Pertussis vaccines: WHO position paper, August 2015—Recommendations. Vaccine.

[2] Bhagat D., Saboui M., Huang G., Domingo F.R., Squires S.G., Salvadori M.I., Li Y.A. (2023). Pertussis epidemiology in Canada, 2005–2019. Can. Commun. Dis. Rep..

[3] European Centre for Disease Prevention and Control (ECDC) (2016). Pertussis—Annual Epidemiological Report 2016.

[4] Gao H., Lau E.H.Y., Cowling B.J. (2022). Waning Immunity After Receipt of Pertussis, Diphtheria, Tetanus, and Polio-Related Vaccines: A Systematic Review and Meta-analysis. J. Infect Dis..

[5] Bagordo F., Grassi T., Savio M., Rota M.C., Baldovin T., Vicentini C., Napolitano F., Trombetta C.M., Gabutti G., Seroepidemiological Study Group (2023). Assessment of Pertussis Underreporting in Italy. J. Clin. Med..

[6] Cherry J.D., Tan T., von König C.-H.W., Forsyth K.D., Thisyakorn U., Greenberg D., Johnson D., Marchant C., Plotkin S. (2012). Clinical definitions of pertussis: Summary of a Global Pertussis Initiative roundtable meeting, February 2011. Clin. Infect Dis..

[7] Ristić M., Radosavljević B., Stojanović V.D., Đilas M., Petrović V. (2018). Performance of the new clinical case definitions of pertussis in pertussis suspected infection and other diagnoses similar to pertussis. PLoS ONE.

[8] Petrović V., Durić P., Stefanović S. (2006). Epidemiological characteristics of pertussis in Vojvodina. Med Pregl..

[9] Petrović R. (1996). Imunizacije.

[10] Pravilnik o imunizaciji i načinu zastite lekovima Službeni glasnik Republike Srbije, broj 88/2018, 11/2018, 14/2018, 45/2018, 48/2018, 58/2018, [4/2018, 6/2021, 52/2021 i 66/2022. (In Serbian). https://pravno-informacioni-sistem.rs/SlGlasnikPortal/eli/rep/sgrs/ministarstva/pravilnik/2018/14/1.

[11] Pravilnik o Programu obavezne i preporučene imunizacije stanovništva protiv određenih zaraznih bolesti Službeni glasnik Republike Srbije, broj 23/2023. (In Serbian). https://pravno-informacioni-sistem.rs/SlGlasnikPortal/eli/rep/sgrs/ministarstva/pravilnik/2023/23/9.

[12] Statistical Office of the Republic of Serbia Census of population, households and dwellings in the Republic of Serbia (1961, 1971, 1981, 1991, 2002, 2011, and 2022). (In Serbian). https://www.stat.gov.rs/en-us/oblasti/popis/.

[13] World Health Organization Immunization Coverage. Department for Vaccines and Biologicals, Immunization, Surveillance and Monitoring System. https://www.who.int/teams/immunization-vaccines-and-biologicals.

[14] Cherry J.D. (1984). The epidemiology of pertussis and pertussis immunization in the United Kingdom and the United States: A comparative study. Curr. Probl. Pediatr..

[15] Bahar E., Shamarina D., Sergerie Y., Mukherjee P. (2022). Descriptive Overview of Pertussis Epidemiology Among Older Adults in Europe During 2010–2020. Infect Dis. Ther..

[16] Clark T.A. (2014). Changing pertussis epidemiology: Everything old is new again. J. Infect. Dis..

[17] Duquet S.A. (2020). Pertussis Resurgence in Europe: Incidence and Epidemiologic Cycles in Immunization Required and Non-Required Countries. Walden Dissertations and Doctoral Studies. 7920. https://scholarworks.waldenu.edu/dissertations/7920.

[18] Guiso N. (2014). Bordetella pertussis: Why is it still circulating?. J. Infect..

[19] Centers for Disease Control and Prevention (2024). Surveillance and Reporting. https://www.cdc.gov/pertussis/surv-reporting.html.

[20] World Health Organization (2023). Global and Regional Immunization Profile: Global. https://immunizationdata.who.int/.

[21] Organization World Health (2023). Diphtheria Tetanus Toxoid and Pertussis (DTP) Vaccination Coverage. https://immunizationdata.who.int/pages/coverage/dtp.html?CODE=Global&ANTIGEN=DTPCV1+DTPCV3&YEAR=.

[22] Meteologos Sačuvano od zaborava—Odbrana novog sada od poplave 1965. Godine. https://www.meteologos.rs/sacuvano-od-zaborava-odbrana-novog-sada-od-poplave-1965-godine/.

[23] Maltezou H.C., Medic S., Cassimos D.C., Effraimidou E., Poland G.A. (2022). Decreasing routine vaccination rates in children in the COVID-19 era. Vaccine.

[24] Macina D., Evans K.E. (2021). Bordetella pertussis in School-Age Children, Adolescents, and Adults: A Systematic Review of Epidemiology, Burden, and Mortality in Asia. Infect. Dis. Ther..

[25] Esposito S., Principi N. (2016). European Society of Clinical Microbiology and Infectious Diseases (ESCMID) Vaccine Study Group (EVASG). Immunization against pertussis in adolescents and adults. Clin. Microbiol. Infect..

[26] Griffith M.M., Fukusumi M., Kobayashi Y., Matsui Y., Nishiki S., Shimbashi R., Morino S., Sunagawa T., Tanaka-Taya K., Matsui T. (2018). Epidemiology of vaccine-preventable diseases in Japan: Considerations for pre-travel advice for the 2019 Rugby World Cup and 2020 Summer Olympic and Paralympic Games. Western Pac. Surveill Response J..

[27] Petrović V., Radosavljević B., Ristić M. (2018). Seroprevalence of pertussis in adult population. Srp. Arh. Celok. Lek..

[28] The Government of the Republic of Serbia COVID-19. http://www.pravno-informacioni-sistem.rs/fp/covid19.

[29] Stein-Zamir C., Shoob H., Abramson N., Brown E.H., Zimmermann Y. (2023). Pertussis outbreak mainly in unvaccinated young children in ultra-orthodox Jewish groups, Jerusalem, Israel 2023. Epidemiol. Infect.

[30] Nian X., Liu H., Cai M., Duan K., Yang X. (2023). Coping Strategies for Pertussis Resurgence. Vaccines.

[31] Jutarnji list (2023). Hripavac se širi, najviše oboljelih u posljednjih 40 godina, HZJZ poslao upozorenje: ‘Imate zakonsku obvezu!’. https://www.jutarnji.hr/vijesti/hrvatska/hripavac-se-siri-najvise-oboljelih-u-posljednjih-40-godina-hzjz-poslao-upozorenje-imate-zakonsku-obvezu-15403366.

[32] European Centre for Disease Prevention and Control Communicable Disease Threats Report. *Week 12*, 17−23 March 2024. https://www.ecdc.europa.eu/sites/default/files/documents/communicable-disease-threats-report-week-12-2024.pdf.

[33] Oliveira S.M., Gonçalves-Pinho M., Freitas A., Guimarães H., Azevedo I. (2018). Trends and Costs of Pertussis Hospitalizations in Portugal, 2000 to 2015: From 0 to 95 years old. Infect Dis..

[34] Plotkin S.A. (2014). The pertussis problem. Clin. Infect. Dis..

[35] Wilk M.M., Borkner L., Misiak A., Curham L., Allen A.C., Mills K.H.G. (2019). Immunization with whole cell but not acellular pertussis vaccines primes CD4 TRM cells that sustain protective immunity against nasal colonization with Bordetella pertussis. Emerg. Microbes Infect..

[36] Esposito S., Stefanelli P., Fry N.K., Fedele G., He Q., Paterson P., Tan T., Knuf M., Rodrigo C., Olivier C.W. (2019). Pertussis Prevention: Reasons for Resurgence, and Differences in the Current Acellular Pertussis Vaccines. Front. Immunol..

[37] Anis E., Moerman L., Ginsberg G., Karakis I., Slater P.E., Warshavsky B., Gosinov R., Grotto I., Marva E. (2018). Did two booster doses for schoolchildren change the epidemiology of pertussis in Israel?. J. Public Health Policy.

[38] Brousseau N., Skowronski D.M., Bellemare D., Amini R., Joffres Y., Clarke Q., Quach C., Rallu F., Hoang L., De Serres G. (2020). Impact of the adolescent pertussis booster dose on the incidence of pertussis in British Columbia and Quebec, Canada. Vaccine.

[39] Skoff T.H., Martin S.W. (2016). Impact of Tetanus Toxoid, Reduced Diphtheria Toxoid, and Acellular Pertussis Vaccinations on Reported Pertussis Cases Among Those 11 to 18 Years of Age in an Era of Waning Pertussis Immunity: A Follow-up Analysis. JAMA Pediatr..

[40] Seppälä E., Kristoffersen A.B., Bøås H., Vestrheim D.F., Greve-Isdahl M., De Blasio B.F., Steens A. (2022). Pertussis epidemiology including direct and indirect effects of the childhood pertussis booster vaccinations, Norway, 1998–2019. Vaccine.

[41] Chen Z., He Q. (2017). Immune persistence after pertussis vaccination. Hum. Vaccin. Immunother..

[42] World Health Organization (2014). Laboratory Manual for the Diagnosis of Whooping Cough Caused by Bordetella Pertussis-Bordetella Parapertussis.

